# A dynamical measure of the black hole mass in a quasar 11 billion years ago

**DOI:** 10.1038/s41586-024-07053-4

**Published:** 2024-01-29

**Authors:** R. Abuter, F. Allouche, A. Amorim, C. Bailet, A. Berdeu, J.-P. Berger, P. Berio, A. Bigioli, O. Boebion, M.-L. Bolzer, H. Bonnet, G. Bourdarot, P. Bourget, W. Brandner, Y. Cao, R. Conzelmann, M. Comin, Y. Clénet, B. Courtney-Barrer, R. Davies, D. Defrère, A. Delboulbé, F. Delplancke-Ströbele, R. Dembet, J. Dexter, P. T. de Zeeuw, A. Drescher, A. Eckart, C. Édouard, F. Eisenhauer, M. Fabricius, H. Feuchtgruber, G. Finger, N. M. Förster Schreiber, P. Garcia, R. Garcia Lopez, F. Gao, E. Gendron, R. Genzel, J. P. Gil, S. Gillessen, T. Gomes, F. Gonté, C. Gouvret, P. Guajardo, S. Guieu, W. Hackenberg, N. Haddad, M. Hartl, X. Haubois, F. Haußmann, G. Heißel, Th. Henning, S. Hippler, S. F. Hönig, M. Horrobin, N. Hubin, E. Jacqmart, L. Jocou, A. Kaufer, P. Kervella, J. Kolb, H. Korhonen, S. Lacour, S. Lagarde, O. Lai, V. Lapeyrère, R. Laugier, J.-B. Le Bouquin, J. Leftley, P. Léna, S. Lewis, D. Liu, B. Lopez, D. Lutz, Y. Magnard, F. Mang, A. Marcotto, D. Maurel, A. Mérand, F. Millour, N. More, H. Netzer, H. Nowacki, M. Nowak, S. Oberti, T. Ott, L. Pallanca, T. Paumard, K. Perraut, G. Perrin, R. Petrov, O. Pfuhl, N. Pourré, S. Rabien, C. Rau, M. Riquelme, S. Robbe-Dubois, S. Rochat, M. Salman, J. Sanchez-Bermudez, D. J. D. Santos, S. Scheithauer, M. Schöller, J. Schubert, N. Schuhler, J. Shangguan, P. Shchekaturov, T. T. Shimizu, A. Sevin, F. Soulez, A. Spang, E. Stadler, A. Sternberg, C. Straubmeier, E. Sturm, C. Sykes, L. J. Tacconi, K. R. W. Tristram, F. Vincent, S. von Fellenberg, S. Uysal, F. Widmann, E. Wieprecht, E. Wiezorrek, J. Woillez, G. Zins

**Affiliations:** 1 European Southern Observatory, Garching, Germany; 2 Université Côte d’Azur, Observatoire de la Côte d’Azur, CNRS, Laboratoire Lagrange, Nice, France; 3 Faculdade de Ciências, Universidade de Lisboa, Lisboa, Portugal; 4 CENTRA – Centro de Astrofísica e Gravitação, Instituto Superior Técnico (IST), Universidade de Lisboa, Lisboa, Portugal; 5 LESIA - Observatoire de Paris, Université PSL, Sorbonne Université, Université Paris Cité, CNRS, Meudon, France; 6 Université Grenoble Alpes, CNRS, IPAG, Grenoble, France; 7 Institute of Astronomy, KU Leuven, Leuven, Belgium; 8 Max Planck Institute for Extraterrestrial Physics, Garching, Germany; 9 Department of Physics, Technical University Munich, Garching, Germany; 10 Univ. Lyon, Univ. Lyon 1, ENS de Lyon, CNRS, Centre de Recherche Astrophysique de Lyon UMR5574, Saint-Genis-Laval, France; 11 European Southern Observatory, Santiago, Chile; 12 Max Planck Institute for Astronomy, Heidelberg, Germany; 13 Research School of Astronomy and Astrophysics, College of Science, Australian National University, Canberra, Australian Capital Territory Australia; 14 Department of Astrophysical & Planetary Sciences, JILA, University of Colorado Boulder, Boulder, CO USA; 15 Leiden University, Leiden, The Netherlands; 16 Max Planck Institute for Radio Astronomy, Bonn, Germany; 17 1st Institute of Physics, University of Cologne, Cologne, Germany; 18 Faculdade de Engenharia, Universidade do Porto, Porto, Portugal; 19 School of Physics, University College Dublin, Belfield, Dublin 4, Ireland; 20 Departments of Physics, University of California, Berkeley, Berkeley, CA USA; 21 Department of Astronomy, University of California, Berkeley, Berkeley, CA USA; 22 Advanced Concepts Team, European Space Agency, TEC-SF, ESTEC, Noordwijk, The Netherlands; 23 School of Physics and Astronomy, University of Southampton, Southampton, UK; 24 School of Physics and Astronomy, Tel Aviv University, Tel Aviv, Israel; 25 Institute of Astronomy, University of Cambridge, Cambridge, UK; 26 Instituto de Astronomía, Universidad Nacional Autónoma de México, Ciudad de México, Mexico; 27 Center for Computational Astrophysics, Flatiron Institute, New York, NY USA

**Keywords:** Galaxies and clusters, Compact astrophysical objects, Early universe

## Abstract

Tight relationships exist in the local Universe between the central stellar properties of galaxies and the mass of their supermassive black hole (SMBH)^1–3^. These suggest that galaxies and black holes co-evolve, with the main regulation mechanism being energetic feedback from accretion onto the black hole during its quasar phase^4–6^. A crucial question is how the relationship between black holes and galaxies evolves with time; a key epoch to examine this relationship is at the peaks of star formation and black hole growth 8–12 billion years ago (redshifts 1–3)^7^. Here we report a dynamical measurement of the mass of the black hole in a luminous quasar at a redshift of 2, with a look back in time of 11 billion years, by spatially resolving the broad-line region (BLR). We detect a 40-μas (0.31-pc) spatial offset between the red and blue photocentres of the Hα line that traces the velocity gradient of a rotating BLR. The flux and differential phase spectra are well reproduced by a thick, moderately inclined disk of gas clouds within the sphere of influence of a central black hole with a mass of 3.2 × 10^8^ solar masses. Molecular gas data reveal a dynamical mass for the host galaxy of 6 × 10^11^ solar masses, which indicates an undermassive black hole accreting at a super-Eddington rate. This suggests a host galaxy that grew faster than the SMBH, indicating a delay between galaxy and black hole formation for some systems.

## Abstract (web summary)

Using the GRAVITY+ instrument, dynamical measurement of the black hole mass in a quasar at a redshift of 2.3 (11 billion years ago) shows how the relationship between galaxies and black holes evolves with time.

## Main

SDSS J092034.17+065718.0 (hereafter J0920) is one of the most luminous quasars at *z* ≈ *2*, making it an attractive target for studies of SMBH growth and its connection to host-galaxy growth. Assuming that the local BLR radius–luminosity relationship^8^ can be applied at high redshift, J0920 is then expected to have a large BLR. Given also its close proximity to a bright star and its bright Hα emission line redshifted into the *K*-band, we observed J0920 with GRAVITY+ (ref. ^9^) at the Very Large Telescope Interferometer (VLTI), an upgrade to GRAVITY^10^, using the new wide-field, off-axis fringe-tracking mode (GRAVITY Wide)^11^.

**Fig. 1:**
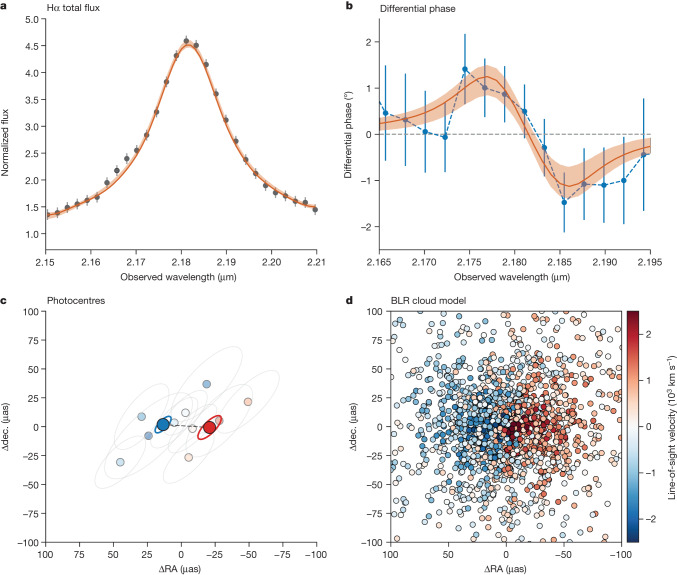
Main BLR observational and modelling results. **a**, Observed GRAVITY+ Hα total flux line profile averaged over the four Unit Telescopes and normalized to the continuum (black points) with 1*σ* error bars. The red curve and shaded region indicate the line profile for our best-fit BLR model and 68th percentile confidence region, respectively. **b**, Differential phase curve across the Hα line averaged over three baselines (blue points) with 1*σ* uncertainties. The red curve and shaded region also show the differential phase for our best-fit BLR model and 68th percentile confidence region, respectively. The distinct S-shape signal is expected for a velocity gradient. **c**, Model-independent photocentres for the central ten wavelength channels (small coloured points). The colour of the points represents the line-of-sight velocity and the grey ellipses show the 68th percentile confidence region. The larger blue and red points with ellipses show the average blueshifted and redshifted photocentres with their 68th percentile confidence regions. **d**, On-sky cloud representation of our best-fit BLR model showing an inclined, rotating, thick disk. As in **c**, the colour represents line-of-sight velocity. Source data

**Extended Data Fig. 1:**
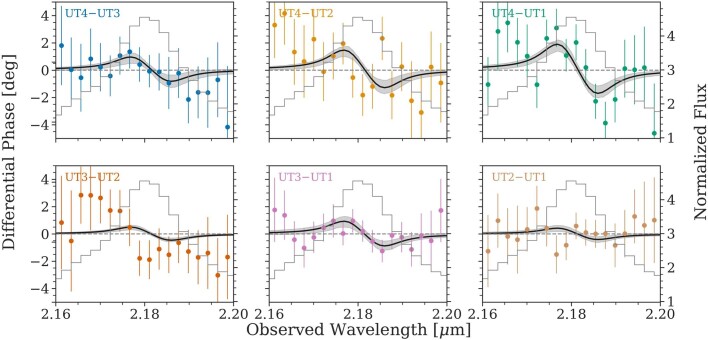
Individual baseline differential phase spectra. Average differential phase spectra for each baseline in the 2.16–2.20-μm region (coloured points with 1*σ* error bars), together with the total flux spectrum (grey line) and best-fit BLR model (black line) with 68th percentile confidence region (shaded region).

From the raw GRAVITY+ frames, we extracted average differential phase curves of J0920 for each of the six baselines. For targets much smaller than the resolution limit, the differential phase is proportional to the displacement of the source photocentre along the baseline. We detect an ‘S-shape’ differential phase signal in the longest baselines (Fig. 1b and Extended Data Fig. 1), characterizing a velocity gradient through the Hα line (Fig. 1a) and suggesting a BLR dominated by rotation, as found in local active galactic nuclei (AGN)^12–15^.

We measure model-independent photocentres for the central ten wavelength channels using all six baselines (Fig. 1c) and observe a global east–west shift from the blue to the red wing of the line, indicative of a velocity gradient. By binning all redshifted and blueshifted channels together, we measure an average separation between the two sides of *D*_photo_ *=* 37 ± 12 μas (0.31 ± 0.10 pc at *z* *=* 2.325), indicating a detection significance of 3–6*σ* (see Methods). Photocentre separations, however, can only provide at best a lower limit on the true BLR size given the unknown geometry, in particular the inclination and opening angle. For these as well as determining the central SMBH mass, detailed kinematic modelling is needed.

We therefore simultaneously fit the six differential phase spectra and total flux spectrum with a kinematic model. The kinematic model consists of a distribution of independent clouds moving within the gravitational potential of the SMBH (Methods). The spectra are well fit by this model (reduced *χ*^2^ = 0.6) and the best fit is shown as the red curve in Fig. 1a,b. Extended Data Table 1 reports the best-fit parameters and their 68th percentile confidence intervals, along with a brief description and the prior used.

We infer a mean Hα-emitting BLR radius of ${R}_{{\rm{BLR}}}={40}_{-13}^{+20}\,\mu$ as (${0.34}_{-0.11}^{+0.17}\,{\rm{pc}}$) within a moderately inclined disk ($i={3{2}^{\circ }}_{-7}^{+8}$) that is oriented on-sky with a position angle ${\rm{PA}}={8{7}^{\circ }}_{-25}^{+19}$. We further infer the BLR half-opening angle to be ${\theta }_{{\rm{o}}}={5{1}^{\circ }}_{-13}^{+11}$, which—combined with the inclination—is consistent with an unobscured quasar. We show an on-sky representation of the best-fit BLR cloud distribution in Fig. 1d.

**Fig. 2:**
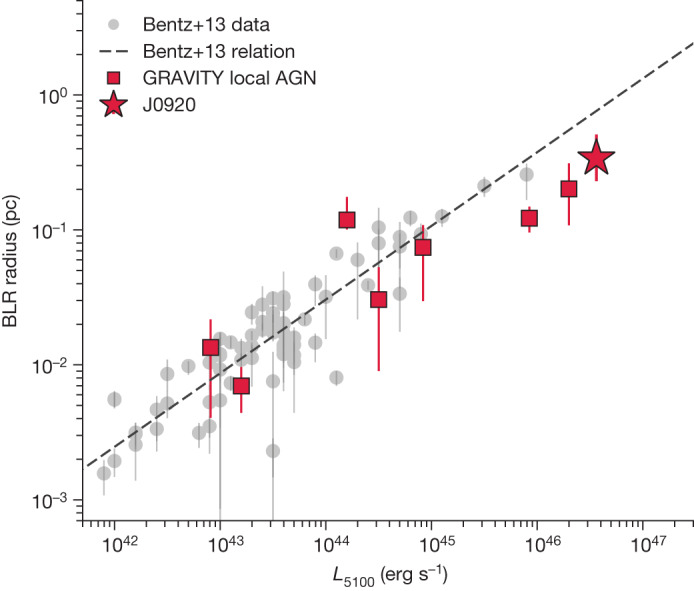
BLR radius–luminosity relation. Empirical correlation between BLR radius and AGN luminosity (as measured by the luminosity at 5,100 ångström). Grey points are reverberation-mapping measurements from ref. ^21^. Moderate luminosity, local AGN measured by GRAVITY (red squares)^12–15^ confirm the reverberation-mapping-based relation (ref. ^11^; dashed line). High-luminosity quasars, including J0920 (red star), indicate a potential deviation from the relation towards smaller radii. All error bars represent 1*σ* uncertainties. Source data

Our measured radius is a factor of 2.25 smaller than what would be inferred from the local Hβ-based radius–luminosity relation^11^ (see Fig. 2 and Methods). Previous studies have actually measured up to a factor of 1.5 larger sizes for the Hα-emitting region compared with Hβ (refs. ^16–18^), as expected for a radially stratified BLR and including optical-depth effects^19^. This would only increase the tension between our spectro-interferometric size and luminosity-based size, although one should bear in mind that the latter is a ‘single-epoch’ method that uses only the linewidth of the BLR and the AGN luminosity and so carries with it a large uncertainty.

However, our smaller size is consistent with the results at lower redshift for the high-luminosity quasars 3C 273 and PDS 456 observed with GRAVITY, as well as reverberation mapping of high-Eddington-ratio AGN^20–23^. Indeed, combining the bolometric luminosity of J0920 (log *L*_Bol_ = 47.2–47.9 erg s^−1^; see Methods) with our GRAVITY+-measured SMBH mass, we find an Eddington ratio *L*_Bol_/*L*_Edd_ = 7–20, which supports previous observations that super-Eddington accreting quasars have smaller BLRs relative to the radius–luminosity relation. More generally, this is further an independent confirmation that super-Eddington quasars exist using a highly accurate SMBH mass. We finally note that the size of J0920 would still correspond to a time lag of about 1,200 days in the observer’s frame, making reverberation-mapping measurements more difficult and substantially longer compared with the few hours needed with GRAVITY+.

Our kinematic modelling infers a SMBH mass of $\log {M}_{{\rm{BH}}}={8.51}_{-0.28}^{+0.27}\,{M}_{\odot }$, which we can compare with mass measurements using the ‘single-epoch’ method from three different emission lines: C IV, Hβ and Hα. On the basis of the C IV linewidth, we determine a mass of log *M*_BH_ ≈ 9.7 *M*_⊙_, or about 1.2 dex larger than our spectro-interferometric result. Comparing the line profiles of C IV and Hα reveals that C IV is both systematically blueshifted by 5,000 km s^−1^ and much broader (full width at half maximum (FWHM) ≈ 8,000 km s^−1^ for C IV compared with 2,500 km s^−1^ for Hα). For J0920, C IV therefore must be tracing a high-velocity, quasar-driven outflow rather than gravitationally bound gas, which reinforces concerns about adopting C IV-based single-epoch masses^24–27^.

We determine a single-epoch Hβ mass of log *M*_BH_ = 9.24 ± 0.47 *M*_⊙_, which is 0.73 dex higher than our measurement from GRAVITY+ data. 0.53 dex of the discrepancy originates in the smaller BLR radius compared with that expected from the local radius–luminosity relation. The remaining discrepancy can be attributed to the *f* scaling factor needed to convert the single-epoch virial product to a black hole mass. This scaling factor has notable systematic uncertainty for individual objects, as it is calibrated as a mean value such that a sample of AGN match the local *M*_BH_–σ_*_ relationship. The single-epoch Hα mass (log *M*_BH_ = 8.94 ± 0.48 *M*_⊙_) is only 0.43 dex larger, again because of the smaller BLR radius. Although the single-epoch and spectro-interferometric Hα mass are in reasonable agreement, our GRAVITY+-based mass has much lower uncertainty, given the ability to self-consistently measure size and mass and not rely on a scaling factor. Finally, we use the formalism of ref. ^28^ to correct the single-epoch Hβ BLR radius for the Eddington ratio and arrive at a BLR radius of 0.2 pc (Methods) and SMBH mass of 8.6 dex, now only 0.1 dex larger than the GRAVITY+-based mass and well within the uncertainties. Consequently, our spectro-interferometric result lends support to the idea that the Eddington ratio is a nuisance factor in the radius–luminosity relation and that the correction proposed in ref. ^28^ may substantially improve single-epoch mass estimates, especially for high-luminosity quasars.

To investigate the host galaxy properties, we observed the CO (3-2) emission line for J0920 with the NOEMA interferometer, which traces the molecular gas in the host galaxy and provides a measure of the galaxy mass, even in the presence of the bright central quasar^29^. We infer a total dynamical mass, $\log ({M}_{{\rm{dyn}}}/{M}_{\odot })={11.77}_{-0.37}^{+0.44}$, and convert to a stellar mass using the average dynamical-to-stellar mass ratio found in *z* ≈ 2 star-forming galaxies^30^, resulting in $\log ({M}_{{\rm{stellar}}}/{M}_{\odot })={11.39}_{-0.39}^{+0.45}$.

**Fig. 3:**
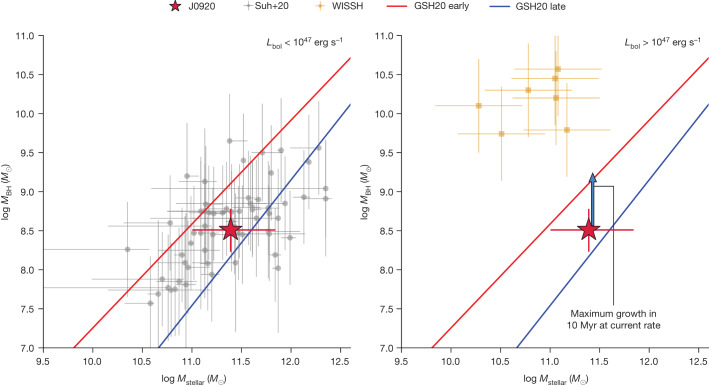
Black hole and host galaxy relation. The location of J0920 in the SMBH mass–stellar mass plane (red star) compared with previously measured *z* ≈ 2 AGN from ref. ^31^ (grey points) and the WISSH survey^32^ (yellow squares). We split the figure into two panels based on the bolometric luminosity of the comparison sample with a cut at *L*_Bol_ = 10^47^ erg s^−1^. Effectively, this places all of the quasars from ref. ^31^ in the left panel with lower luminosities and all of the WISSH quasars in the right panel with high luminosities. Although J0920 has *L*_Bol_ > 10^47^ erg s^−1^, we still plot it in both panels for comparison. GRAVITY+ provides a greatly improved constraint on the SMBH mass. J0920 clearly lies well below the high-luminosity WISSH quasars and within the population of the ref. ^31^ sample, showing the unique nature of J0920. Compared with recent local scaling relations^36^, J0920 is off the early-type galaxy relation (red line) and near the late-type galaxy relation (blue line). Given the SMBH accretion rate of J0920, it should shift directly up towards the early-type relation (blue arrow in right panel) and indicates that it is in a state of rapid SMBH growth at present. All error bars represent 1*σ* uncertainties. Source data

In Fig. 3, we show J0920 on the *M*_BH_–*M*_stellar_ plane for *z* *≈* 2. The two panels of Fig. 3 split our comparison samples based on bolometric luminosity, with high-luminosity (*L*_Bol_ > 10^47^ erg s^−1^) quasars on the right and lower-luminosity ones on the left. For lower-luminosity quasars, we use a sample of *z* *=* 1.5*–*2.5 galaxies from ref. ^31^ (grey points; left panel) for which both *M*_BH_ and *M*_stellar_ have been measured. *M*_BH_ values for this sample were determined through the single-epoch method using the Hα, Hβ or Mg II broad emission line. Despite its higher luminosity, J0920 sits within the population of this sample. For high-luminosity quasars, we use the WISSH survey^32^ (yellow points; right panel) quasars with published CO line measurements to convert them to *M*_stellar_ in a similar way as for J0920 (ref. ^33^). *M*_BH_ values are based on single-epoch measurements with either the Hβ line^34^ or the C IV line^35^. J0920 lies well below the WISSH quasars, with a SMBH mass approximately 100 times smaller, despite a comparable host galaxy mass and AGN luminosity. We point out that about 0.7 dex of the discrepancy can be alleviated if the deviation of the Hβ-based radius–luminosity relation at high luminosity or Eddington ratio holds true. Also, the C IV-based masses may be greatly overestimated if, as for J0920, outflowing gas dominates the C IV linewidth. However, this only applies to half of the WISSH quasars. Even with these corrections, J0920 seems to have an undermassive SMBH, given its luminosity and stellar mass that are more in line with more moderate-luminosity quasars.

We further compare J0920 with the *M*_BH_–*M*_stellar_ local scaling relations, using a recent measurement of the relations for early-type (red line, Fig. 3) and late-type galaxies^36^ (blue line, Fig. 3). J0920 lies firmly on the late-type galaxy relation and well below the early-type galaxy relation, consistent with a recent study of thousands of local AGN, which found that undermassive SMBHs typically have high accretion rates^37^. Massive, gas-rich galaxies at *z* ≈ 2 are thought to be the progenitors of massive ellipticals in the local Universe^38^. These objects should therefore evolve onto the early-type relation in Fig. 3 by *z* = 0. J0920 would require more than a factor of ten growth in black hole mass and little growth in host galaxy stellar mass to reach this relation. The SMBH, however, is—at present—accreting material at an exceptionally fast rate of 30–140 *M*_⊙_ year^−1^, depending on the specific bolometric correction (see Methods). Using an accretion rate of 85 *M*_⊙_ year^−1^, we show as a blue arrow in Fig. 3 the position of J0920 after 10^7^ years, which corresponds to the expected quasar lifetime^39^. J0920 would evolve directly onto the local early-type galaxy relation. However, it is highly unlikely that the SMBH in J0920 would continue accreting material at such high super-Eddington rates for such a long time. Rather, several longer (approximately 10^8^ years) quasar episodes at more moderate Eddington ratios would be required to reach the local early-type relation.

Some large-scale cosmological simulations predict that galaxies in the early Universe outgrow their SMBHs and attribute it to black hole growth in lower-mass galaxies being inefficient^40,41^. One reason for this may be strong supernovae feedback, in which gas is quickly expelled from the central regions before it can reach the SMBH and only when galaxies become massive enough to retain a nuclear gas reservoir against supernovae feedback do SMBHs begin to rapidly grow. This seems to be the likely scenario driving the evolution of J0920 given its current observed black hole mass, stellar mass and black hole accretion rate. Whether this is the dominant mode of SMBH–galaxy co-evolution will only be revealed with more high-precision SMBH mass measurements.

## Methods

### Target selection

We selected J0920 from the Million Quasars Catalog^42^ after associating each quasar to the nearest stars from the 2MASS Point Source Catalog. J0920 itself is detected in the 2MASS Point Source Catalog with a *K*-band Vega magnitude of 15.1 and is located 12.7 arcsec away from the *K* = 10.4 star, 2MASS 09203423+0657053. The initial redshift for J0920 (*z* = 2.30) was measured as part of the LAMOST quasar survey^43^.

### GRAVITY+ observations and data reduction

We observed J0920 at the VLTI with GRAVITY+ in the new GRAVITY Wide mode as part of an Open Time Service Mode programme (PID: 110.2427, PI: T. Shimizu). We used the medium-resolution (*R* ≈ 500) grating of the science channel spectrograph with combined polarization and the 300-Hz fringe-tracking frequency. As the fringe-tracking object, we used the star 2MASS 09203423+0657053. Science exposures consisted of four 100-s detector integrations (DIT = 100 s, NDIT = 4). A normal observing block was a sequence of six science exposures followed by a sky exposure, in which the science and fringe-tracking fibres were moved 2″ in right ascension and declination away from their nominal position.

Observing blocks were executed over four nights on 9 December 2022, 6 January 2023, 10 January 2023 and 11 January 2023 under excellent weather conditions (average seeing = 0.48″, average coherence time = 11.3 ms). We obtained in total 32 exposures (128 DITs), resulting in an on-source integration time of 3.56 h. However, on 6 January 2023, the UT4 science channel fibre was positioned off the quasar. Therefore, only the three non-UT4 baselines from this night are used for further analysis.

We first used the standard GRAVITY pipeline^44^ (v1.4.2) to reduce all raw files up to the application of the pixel-to-visibility matrix (P2VM). This means that the pipeline performed the bias and sky subtraction, flat fielding, wavelength calibration and spectral extraction steps. Application of the P2VM converts the pixel detector counts into complex visibilities taking into account all instrumental effects, including relative throughput, coherence, phase shift and cross-talk. This results in four complex visibility spectra per baseline per exposure covering the 1.97–2.48-μm wavelength range.

At this point, we proceeded to process the intermediate products (that is, dualscip2vmred.fits files) with our own scripts. This was meant to mitigate potential effects related to the unique situation in which most of the signal is within the emission line and not the continuum. We first measured the coherent flux within the line by summing the spectral channels between 2.17 and 2.19 μm, covering roughly the FWHM of the line. We removed frames in which the integrated emission line coherent flux was fewer than 10^3.5^ counts. This limit was chosen on the basis of the integrated emission line coherent flux measured on the UT4 baselines from 6 January 2023. On this night, the science channel fibre for UT4 was not positioned on the quasar, so any measured coherent flux is noise. Frames showed a maximum emission line coherent flux of 10^3.5^ counts, which we then chose as our threshold for accepting frames on other nights. For the selected frames, we first subtracted the pipeline-measured self-referenced phases, which are a third-degree polynomial fit to the whole wavelength range of each visibility spectrum. We then cut out the 2.10–2.26-μm region and measured and subtracted a second third-degree polynomial to the visibility phases to remove any remaining residual instrumental phase and produce the differential phase spectra. To avoid large outliers influencing the fit, we used the FittingWithOutlierRemoval function in the astropy.modeling module^45^ to iteratively perform fits and at each step remove all channels more than 3*σ* away from the previous best fit. The stopping criterion is then when either no channels are thrown away or five iterations is reached. On average, only 1–2 iterations were needed per baseline. Finally, we averaged over time all phase-flattened complex visibilities per baseline and calculated the resulting average differential phase spectra. Phase uncertainties per spectral channel were measured with the method described in ref. ^46^. At high signal-to-noise ratio, this simply reduces to the standard error of the mean. The averaged differential phase spectra through the inner part of the Hα line are shown in Extended Data Fig. 1.

To calibrate the total flux spectrum, we used the data from 9 December 2022, in which the observing blocks were executed directly after the observation of a bright binary star pair calibrator with GRAVITY Wide. We reduced the calibrator data using the same pipeline and divided the spectra of J0920 by the calibrator spectra for each telescope to remove the atmospheric and instrumental response. We then averaged the four spectra to produce a single total flux spectrum for J0920. As the differential phase and BLR modelling is only sensitive to the line-to-continuum ratio, we measured the underlying continuum by fitting a second-degree polynomial to the 2.05–2.10-μm and 2.25–2.35-μm regions. The best-fit continuum was then divided out of the flux spectrum for the final normalized line profile. The line profile is shown in Extended Data Fig. 1 and Fig. 1a. As uncertainty on the line profile, we measure the root-mean-square variation in the continuum-fitted regions, finding a value of 0.05. We multiply this by a factor of 2 to conservatively account for systematic effects.

### Photocentre measurement

The first analysis performed on the GRAVITY+ differential phases and line profile is the measurement of model-independent photocentres as a function of wavelength/velocity. We use the same procedure as in previous AGN observations^12–14^ and briefly describe it here. In the marginally resolved limit, the differential phase, ΔΦ_*ij*_ = −2π*f*_line_(*u*_*j*_*x*_*i*_ *+* *v*_*j*_*y*_*i*_), in which *i* runs across wavelength and *j* runs across baselines, (*u*_*j*_, *v*_*j*_) are the projected baseline coordinates and (*x*_*i*_, *y*_*i*_) the on-sky photocentre coordinates for each spectral channel and *f*_line_ = *f*_*i*_/(1 + *f*_*i*_), in which *f*_*i*_ is the line intensity as a fraction of the continuum. We use the emcee package^47^ to perform Markov chain Monte Carlo sampling to fit for (*x*_*i*_, *y*_*i*_) of the central ten spectral channels across the Hα line and sample the posterior. We use the median of each marginalized posterior as our best photocentre positions and determine the uncertainty by fitting a 2D Gaussian to the joint posterior of each (*x*_*i*_, *y*_*i*_) pair. The best-fit photocentres and uncertainties are shown in Fig. 1c, in which we clearly see redshifted and blueshifted positions on opposite sides of the central channel along a line in the east–west direction.

We also measure an average red–blue offset, which we term the ‘2-pole’ model. To do this, we first set the central wavelength (2.182 μm) to define which channels are redshifted and which are blueshifted. The model then assumes that all redshifted channels share the same photocentre coordinate (*x*_red_*,* *y*_red_) and all blueshifted channels share the same photocentre coordinate (*x*_blue_, *y*_blue_). We further include a systematic shift of the BLR shared by all channels, (*x*_off_*,* *y*_off_). The fitting is performed in the same way as above but with only two photocentre coordinate pairs as the free parameters. We find (*x*_blue_, *y*_blue_) = (13.6, 1.6) ± (5.8, 7.0) μas and (*x*_red_, *y*_red_) = (−20.6, −0.6) ± (8.6, 10.1) μas, which are shown as the large points in Fig. 1c. The *χ*^2^_2-pole_ = 38.8.

Finally, we perform a third fit now assuming that all spectral channels lie at the same photocentre (*x*_null_, *y*_null_) and the BLR is completely unresolved. This results in either differential phase spectra equal to 0 at all wavelengths (if *x*_null_ = *y*_null_ = 0) or differential phase spectra with the same shape as the emission line profile. We find (*x*_null_, *y*_null_) = (3.3, −3.6) ± (3.8, 9.8) μas with *χ*^2^_null_ = 54.3.

We use an *F-*test to compare the ‘2-pole’ and null model and determine whether the ‘2-pole’ model gives a notably better fit. The *F* statistic is $\frac{\left(\frac{{\chi }_{\text{null}}^{2}-{\chi }_{2-\text{pole}}^{2}}{{p}_{2-\text{pole}}-{p}_{\text{null}}}\right)}{\left(\frac{{\chi }_{2-\text{pole}}^{2}}{n-{p}_{2-\text{pole}}}\right)}$, in which the *χ*^2^ are the total *χ*^2^ from each fit, *p* are the number of parameters for each model and *n* is the number of data points used in the fit. We calculate *F* *=* 5.41, which corresponds to a *P-*value of 10^−9^ and a significance of 6*σ* to reject the null model.

To test for systematics, we downloaded 22 archival calibrator observations in the GRAVITY Wide mode, which results in 664 individual frames that have signal-to-noise ratio comparable with J0920. These data should have zero differential phase because they are single stars and therefore allow for testing while including systematics. We processed the calibrator data in the same manner as J0920 and measured the average redshifted and blueshifted positions using the same wavelength channels and emission line profile. We fit the distribution of red–blue separations with a truncated Gaussian, finding a standard deviation of 12 μas. Given the measured separation for J0920 of 37 μas, this indicates a significance of at least 3*σ*. We consider this a lower limit because we did not specifically test how often the broader S-shape signal of J0920 occurs. Rather, it is likely that many of the non-zero red–blue separations measured in the calibrator data are caused by narrow noise spikes.

### BLR modelling

Our primary analysis centres on modelling the BLR structure and kinematics using the GRAVITY+-observed differential phase and total flux spectra. We refrain from a detailed description of the model and fitting procedure, as this has been outlined in several previous publications^12–14^. In general, we model the BLR as a set of independent, non-collisional clouds solely under the gravitational influence of the central SMBH. The model very closely follows the one used to fit reverberation-mapping data^48,49^, with the main adjustment to output differential phases instead of light curves^50^. Although the model contains several parameters to introduce deviations away from the axisymmetric Keplerian model, we choose to omit those and only use the minimal number of parameters able to best describe our data. The fitted model therefore contains 11 free parameters: *R*_BLR_, *β*, PA, *θ*_0_, *i*, *F*, *M*_BH_, *f*_peak_, *λ*_emit_, *x*_0_ and *y*_0_. A brief description of each parameter along with the prior distributions used in the fitting is given in Extended Data Table 1.

We fit the model to both the total flux spectrum and six baseline-averaged differential spectra. We fit only the central 2.15–2.21-μm region with the highest signal-to-noise ratio but note that fits over the entire 2.1–2.26-μm wavelength range do not produce notably different results. We used the dynesty package^51^ (v2.1), which performs dynamic nested sampling^52^ to sample the potentially complicated posterior. We used multi-ellipsoidal decomposition to bound the target posterior distribution (bound = ‘multi’*)* and the random walk sampling method. Sampling was done with 2,000 live points and we chose to stop sampling once the iterative change in the logarithm of the evidence is less than 0.01 (dlogz_init = 0.01).

**Extended Data Fig. 2:**
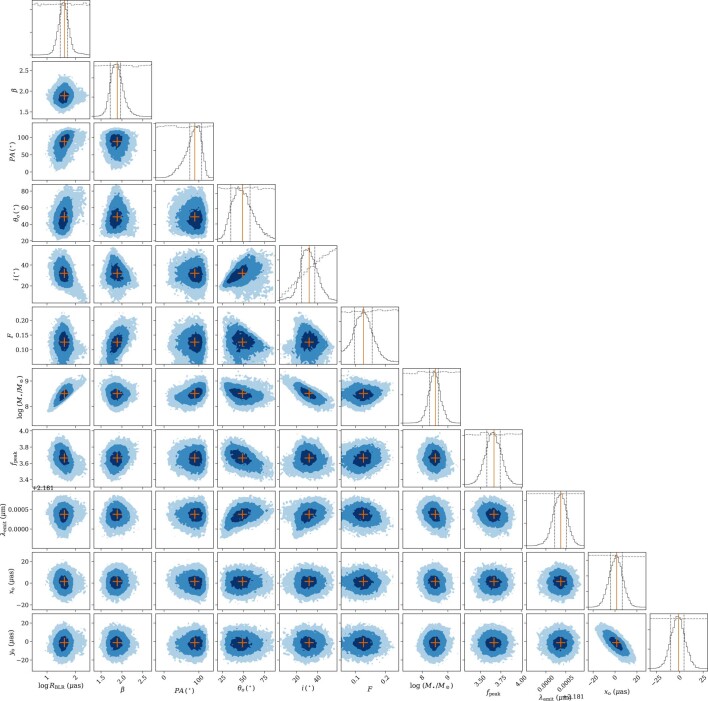
Corner plot of the 2D and 1D posterior distributions for the BLR fit. We plot the 2D joint and 1D marginalized posterior distribution for each parameter of the BLR model used to fit the differential phase and flux spectra. Blue shaded contours represent the 1*σ*, 2*σ* and 3*σ* regions and the orange crosses are the median values that are also reported in Extended Data Table 1. The dotted lines in the 1D posteriors indicate the 16th and 84th percentiles. The orange lines are again the median value. The dashed grey lines show a sampling of the priors used in the fitting, which are listed in Extended Data Table 1.

In Extended Data Fig. 2, we plot the 2D joint and 1D marginalized posterior distributions. The posteriors are well sampled and largely show symmetric, Gaussian-shaped posteriors. We report in Extended Data Table 1 the medians of each 1D marginalized posterior distribution and as uncertainties the 68th percentile confidence interval. We further plot the prior distributions for each parameter used in the modelling with the 1D marginalized posterior distributions. The posteriors have substantially shifted and/or narrowed from the initial prior, showing that the data well constrain each parameter.

To test for potential systematic errors, we fit the data with the full kinematic model including all asymmetric parameters and radial motion. Even though this adds another seven extra free parameters, the reduced chi-square is not improved compared with the simpler axisymmetric model and the posteriors of the extra parameters largely indicate they are unconstrained with distributions similar to the input priors. An advantage of dynesty is the measurement of the Bayesian evidence (*Z*), which can be used to compare models. We find ln(*Z*_sym_) = −333 for the axisymmetric model and ln(*Z*_full_) = −332 for the full model. The ratio of the evidences, or Bayes factor, then quantifies the support for one model over the other. We calculate a Bayes factor, *Z*_full_/*Z*_sym_ = 2.7, which indicates weak support for the full model over the simpler, axisymmetric model. We further note that the uncertainties on all of the original parameters do not markedly increase. However, the median of the posterior for the SMBH mass does slightly increase from log *M*_BH_ = 8.51 to 8.67. This shift is within the 1*σ* uncertainty but suggests a further potential systematic uncertainty. We therefore add in quadrature 0.16 dex to the statistical uncertainty of the black hole mass, resulting in a final uncertainty of 0.27 and 0.28 dex for the upper and lower uncertainties, respectively.

### APO/TripleSpec observations and data reduction

We observed J0920 with the TripleSpec instrument at Apache Point Observatory (APO) for 56 min on 21 December 2021 with a slit width of 1.1″, providing a spectral resolution of 3,181  over the *H* and *K* wavelength bands.

### APO/TripleSpec emission line measurements

**Extended Data Fig. 3:**
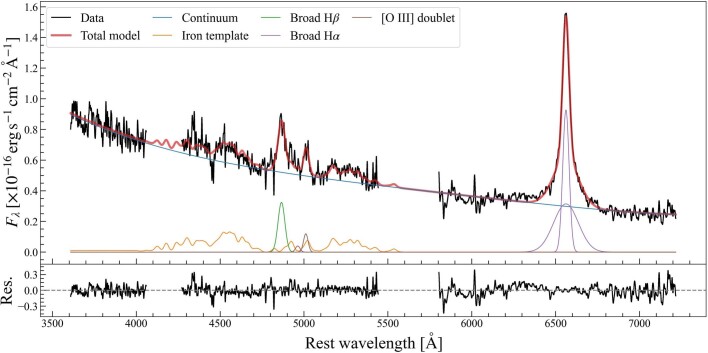
APO/TripleSpec observed *H*+*K* spectrum and spectral decomposition. The top panel shows our flux-calibrated APO/TripleSpec spectrum (black line), together with our best-fit model (red line). The model consists of the following components: fourth-order polynomial for the continuum (blue line), Fe II template (orange line), Hβ Gaussian emission line (green line), [O III] Gaussian emission lines (brown lines) and two Hα Gaussian components (purple lines). The best fit matches the data very well with relative residuals (lower panel) below 20%. The data and residuals are smoothed by a Gaussian kernel with the standard deviation of three channels for clarity of display, whereas the fitting is conducted with the originally reduced data.

The TripleSpec spectrum provides the rest-frame optical spectrum of J0920 at much higher spectral resolution compared with GRAVITY+ and covers the Hβ-[O III] region. This provides an opportunity to compare our spatially resolved BLR size and dynamically measured SMBH mass with those inferred from the single-epoch method. We first scaled the *H*–*K* band spectrum to match the *K*-band magnitude of J0920 from the 2MASS Point Source Catalog (*K* = 15). We simultaneously fit the continuum, Fe II features, Hα, Hβ and [O III] doublet and adopt a fourth-order polynomial to describe the continuum combined with the Fe II template from ref. ^53^. To model the [O III] doublet, we use a single Gaussian component while fixing the [O III] doublet flux ratio to the theoretical value of 2.98 (ref. ^54^) and tying the velocity and linewidth together for the two components of the doublet. Although for Hβ we use only a single Gaussian component, for Hα, we found that we needed two Gaussian components to adequately fit the line but note that we do not consider each component to be tracing different physical components of the emission. Rather, the line profile is probably better described by a Lorentzian shape. We find very good agreement between the TripleSpec line profile and the GRAVITY+ line profile after degrading the TripleSpec line profile to the spectral resolution of GRAVITY+, indicating that we are not seeing extra, more extended narrow line emission in the much larger aperture of TripleSpec. In Extended Data Table 2, we list the best-fit parameters of our spectral decomposition as well as the derived properties and show in Extended Data Fig. 3 the best-fit model and decomposition, along with the residuals. The fitting residuals are about 10^17^ erg s^−1^ cm^−2^ ångström^−1^ around 5,000 ångström and 0.7 × 10^17^ erg s^−1^ cm^−2^ ångström^−1^ around 6,500 ångström. The uncertainties of the measured quantities are derived by refitting the spectra after adding Gaussian noise with a standard deviation equal to the fitting residual at the corresponding wavelength.

In Extended Data Table 2, EW is defined as the equivalent width, *R*_Fe_ is defined as the ratio of Fe II template equivalent width within 4,434–4,684 ångström to Hβ equivalent width and *L*_5100_ is the monochromatic luminosity at rest-frame wavelength 5,100 ångström. We first calculate the bolometric luminosity (*L*_Bol_) using the empirical relation from ref. ^55^, which is based on an average luminosity-dependent quasar spectral energy distribution. The bolometric correction here is about 5 and already placing J0920 well into the super-Eddington regime. Therefore, we also estimate the bolometric luminosity under the slim disk accretion model, which is theorized to be applicable for highly accreting black holes. We use equation (3) from ref. ^56^ to determine a bolometric correction of roughly 23. The bolometric luminosities for both corrections are listed in Extended Data Table 2. From the bolometric luminosity, we estimate a mass accretion rate onto the SMBH of $\mathop{M}\limits^{.}$ = *L*_Bol_/*ηc*^2^ *M*_⊙_ year^−1^ using a standard conversion efficiency, *η* = 0.1.

### Comparison with single-epoch estimates

#### C IV

**Extended Data Fig. 4:**
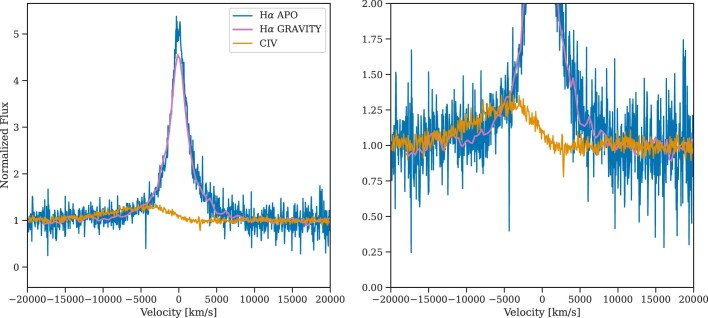
Comparison between C IV and Hα line profiles. We compare the continuum-normalized line profiles of C IV from the LAMOST quasar survey (orange) to both of our Hα line profiles from GRAVITY (purple) and APO/TripleSpec (blue). Wavelengths were converted to velocities using the measured redshift of Hα (*z* *=* 2.325). C IV shows both a systematic blueshift of about 7,000 km s^−1^ and increased linewidth compared with Hα, along with a heavy skew to blueshifted velocities. C IV therefore is probably dominated by outflowing gas and not the virial motion of the BLR.

Our first comparison is with the C IV-based mass estimate, which was measured for the LAMOST QSO Catalog^43^. The reported redshift and FWHM of the C IV line are 2.3015 and 8,013 km s^−1^, respectively. They use the C IV radius–luminosity relation from ref. ^57^ to determine a SMBH mass of 10^9.7^ *M*_⊙_. Compared with our Hα measurements, the redshift is off by 0.0235 (7,050 km s^−1^), the FWHM is a factor of approximately 3 larger and the SMBH mass is 1.2 dex larger. In Extended Data Fig. 4, we compare the line profiles of C IV and Hα using *z* = 2.325 to convert wavelengths into velocities. This shows clearly the substantial blueshift of the C IV line relative to the systemic velocity of Hα, as well as the increased linewidth. Because single-epoch masses scale with the FWHM^2^, the factor of 3 larger FWHM mostly explains the factor of 15 increase in the SMBH mass. Beyond the systematic blueshift of C IV, the line shape is also heavily skewed towards large blueshifted velocities. All of these properties point to C IV emission being dominated by non-virial motions and probably originating in a strong outflow^58,59^. Previous surveys of high-redshift quasars have reported strong correlations between C IV blueshift and FWHM and an anticorrelation between C IV blueshift and Hα FWHM^25,60^, which leads to C IV overestimating the SMBH mass. In fact, ref. ^60^ provides a correction to C IV-based masses based on the blueshift and FWHM of C IV. Applying this (see equations (4) and (6) of ref. ^60^) to J0920, we calculate a corrected C IV SMBH mass of 10^8.7^ *M*_⊙_, which is much closer to our dynamically based mass.

#### Hα and Hβ

We further compare our GRAVITY+-based BLR size and SMBH mass with the single-epoch sizes and masses inferred from the Hα and Hβ relations. We first calculate *R*_BLR_ from an extrapolation of the ‘Clean2’ Hβ radius–luminosity relation from ref. ^8^: log *R*_BLR_ = 1.56 + 0.546log(*L*_5100_/10^44^ erg s^−1^) (light-days). This gives *R*_BLR_ = 907 light-days or 0.765 pc, which is a factor of 2.25 times larger than our spatially resolved measurement. This radius–luminosity relation has a scatter of 0.13 dex, so our smaller size is 1.65*σ* away from the best fit. If the Hα-emitting region is larger than the Hβ-emitting region, as observationally found from reverberation-mapping studies^17,18,57^ and expected from BLR photoionization models^19^, then our BLR size is even more discrepant from the radius–luminosity relation size.

We estimate the Hβ single-epoch SMBH mass using the standard virial relation *M*_BH_ = *f*(*R*_BLR_Δ*v*^2^/G), in which Δ*v* is a measure of the linewidth and *f* is a scale factor that accounts for the orientation and geometry of the BLR. For Δ*v*, we choose to use the Hβ FWHM. We further use log*<f>* = 0.05 ± 0.12, which was determined empirically by fitting the Hβ FWHM-based black hole masses onto the local *M*_BH_–σ_*_ relation^61^. The intrinsic scatter associated with the Hβ single-epoch calibration is measured to be 0.43 dex (ref. ^57^). The Hβ single-epoch black hole mass is then log *M*_BH_ *=* 9.24 ± 0.47, which is 0.73 dex larger than our dynamical measurement. Taking into account the expected factor of 1.5 larger sizes for the Hα-emitting region^17^, then 0.53 dex of the discrepancy can be explained by the much smaller BLR we measure with GRAVITY+. The remaining 0.2 dex can then be explained by scatter in BLR inclination and geometry, leading to variations in individual *f* scale factors.

We use equation (1) from ref. ^62^ to calculate the Hα single-epoch mass, which was calibrated off the Hβ radius–luminosity relation and a correlation between the FWHM of Hβ and Hα and between *L*_5100_ and *L*_Hα_: log(*M*_BH_/*M*_⊙_) = log(*f*) + 6.57 + 0.47log(*L*_Hα_/10^42^ erg s^−1^) + 2.06log(FWHM_Hα_/1,000 km s^−1^). Using the same *f* scaling factor as before, we find log(*M*_BH_/*M*_⊙_) = 8.94 ± 0.48, which is only 0.43 dex larger than our dynamical measurement and within the uncertainties of the single-epoch measurement. This can then be fully explained by the smaller BLR size we measure compared with the expectation from the radius–luminosity relation.

Deviations from the standard radius–luminosity relation have been seen and explored before, with most of the scatter leading to smaller sizes for a given AGN luminosity^21–23,56,63^. Reference ^56^ found that the offset from the radius–luminosity relation was correlated with the Eddington ratio. After gathering a large sample of reverberation-mapping measurements for high-Eddington-ratio targets through the Super-Eddington Accreting Massive Black Hole (SEAMBH) survey, ref. ^28^ proposed a new parameterization of the radius–luminosity relation including *R*_Fe_, the flux ratio of Fe II features between 4,434 and 4,684 ångström and broad Hβ. The Eddington ratio has been shown to be the dominant property driving variations in *R*_Fe_ between AGN^64–66^ and therefore including *R*_Fe_ implicitly adds a second property determining the BLR size beyond the AGN luminosity. The new parameterization is log *R*_BLR_ = 1.65 + 0.45log(*L*_5100_/10^44^ erg s^−1^) − 0.35*R*_Fe_ (light-days). With this, we calculate an Eddington-ratio-corrected BLR size of 237 light-days or 0.2 pc, a factor of 1.7 smaller than our GRAVITY+-measured size. This then leads to a log(*M*_BH_/*M*_⊙_) = 8.66 using the same *f* scaling factor as above, the closest ‘single-epoch’ estimate to our dynamical measurement. Although J0920 is only one object, this certainly adds to the evidence that the BLR size is related to the Eddington ratio of the SMBH and thus should be taken into account for SMBH mass measurements.

#### NOEMA observations and data reduction

To complement our GRAVITY+ observations and examine the host galaxy of J0920, we observed J0920 with the IRAM Northern Extended Millimeter Array (NOEMA) as part of a larger pilot survey of *z* *≈* 2 quasars (ID: S22CE, PI: J. Shangguan) on 12 June and 18 September 2022 in D configuration. The total on-source time was 3.9 h with ten antennae. We set the phase centre to the known coordinates of J0920 (RA = 09 h 20 min 34.171 s, dec. = 06° 57′ 18.019″) and used the PolyFiX correlator with a total bandwidth of 15.5 GHz. With a tuning frequency of 104.7867 GHz, we placed the redshifted CO (3-2) molecular gas line (*ν*_rest_ = 345.7960 GHz, *ν*_obs_ = 103.99 GHz) into the upper sideband.

The sources J0923+392, J2010+723, J0906+015 and J0851+202 were used as flux calibrators and J0906+015 and J0851+202 were used for phase calibration. Observations were taken under average weather conditions with precipitable water vapour of 4–10 mm. We reduced and calibrated the data with the CLIC package of GILDAS to produce the final (*u*, *v*) tables.

The (*u*, *v*) tables were then imaged with the MAPPING package of GILDAS using the Högbom CLEAN algorithm. We adopted natural weighting of the visibilities, resulting in a synthesized beam of 4.7″ × 3.2″. We ran CLEAN until the maximum of the absolute value of the residual map was lower than 0.5*σ*, with *σ* the root-mean-square noise of the cleaned image and used a circular support mask with diameter 18″ centred on J0920. We then resampled the spectral axis to 40 km s^−1^ bins, achieving a root-mean-square noise of 0.388 mJy beam^−1^.

#### Host galaxy properties

**Extended Data Fig. 5:**
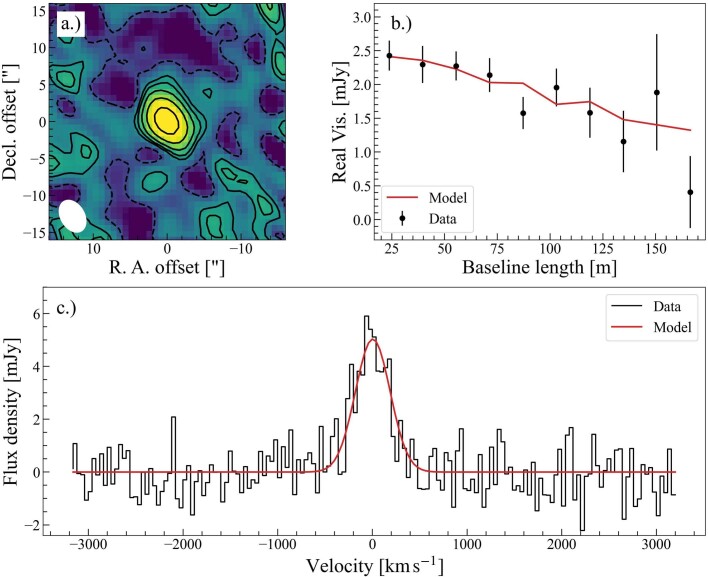
NOEMA CO (3-2) data and analysis. **a**, Moment 0 map of J0920 using the channels spanning −700 to 700 km s^−1^ around the expected location of the CO (3-2) line. The contours are (−1, 1, 2, 4, 8, 16) times the root-mean-square noise level, with the −1*σ* level in the dashed line. The synthesized beam (4.7″ × 3.2″) is shown in the lower-left corner. **b**, Average real part of the visibilities as a function of baseline length (black points) showing decreasing visibility with increasing baseline with 1*σ* error bars. This indicates that J0920 is extended even with the relatively large beam size. The red line is a fit using an elliptical exponential disk model in which we find an effective radius of 8.23 kpc. **c**, Integrated spectrum within the 1*σ* contour shown in panel **a** showing the detection of the CO (3-2) line. We fit the line with a single Gaussian (red line), finding a FWHM of 432 ± 42 km s^−1^ and use this with the effective radius determined in panel **b** to estimate the dynamical mass of J0920 and place it on the SMBH–galaxy scaling relation (see main text).

In Extended Data Fig. 5a, we show the 0th moment image of the cube generated between −700 and 700 km s^−1^ around the expected location of the CO (3-2) line. We clearly detect J0920 with a maximum signal-to-noise ratio of >20 and visual comparison of the image with the synthesized beam suggests that J0920 is extended especially in the north–south direction. To test this and measure a CO size, we used UVFIT in GILDAS to fit the visibilities directly with an elliptical exponential disk model. Extended Data Fig. 5b shows the visibilities as a function of baseline length together with our best-fit model. The clear decrease with baseline length is indicative of a partially resolved source. Our best-fit disk model fits the data well and confirms the resolved nature. A Gaussian disk model provides a nearly equally good fit and the same effective radius as the exponential disk considering the uncertainty. We prefer to adopt the results with the exponential disk model to facilitate estimating the dynamical mass of the host galaxy using the empirical relation of ref. ^29^.

We measure an effective radius of the disk, *R*_e_ = 8.23 ± 1.53 kpc, a position angle on sky of 90.0° ± 0.4° and an axis ratio of 1.66 ± 0.8. This places J0920 at the upper envelope of the size–mass relation for its redshift and firmly within the late-type galaxy population^67^, under the assumption that the molecular gas disk traces the stellar disk.

To measure the CO (3-2) flux and linewidth, we extracted a 1D spectrum by integrating the cube within the 1*σ* contour of the 0th moment map. We plot the resulting spectrum in Extended Data Fig. 5c, which shows clearly the CO (3-2) line. We fit the line from the integrated spectrum with a single Gaussian component, finding a redshift of 2.3253 ± 0.0002 (very similar to the Hα redshift), integrated flux of 2.330 ± 0.162 Jy km s^−1^ and a FWHM of 432 ± 42 km s^−1^.

Reference ^29^ provides empirical relations between the dynamical mass of a system and unresolved, integrated line properties based on spatially resolved kinematic modelling of *z* ≈ 6 quasar host galaxies. Here we use equation (15), which assumes that robust measurements of the line FWHM and radial extent of the galaxy have been made, as in the case of J0920: ${M}_{{\rm{dyn}}}={1.9}_{-0.8}^{+1.5}({}_{-1.3}^{+1.1})\times {10}^{5}{\left({\rm{FWHM}}\right)}^{2}{R}_{{\rm{e}}}\,({M}_{\odot })$, in which FWHM is in km s^−1^ and *R*_e_ is in kpc. For J0920, we find $\log ({M}_{{\rm{dyn}}}/{M}_{\odot })={11.77}_{-0.37}^{+0.44}$, in which the uncertainties are a combination of the measurement errors of the line and the statistical (first set of uncertainties in the equation) and systematic uncertainties (second set of uncertainties in the equation) of the empirical relation.

To infer the stellar mass, we use the empirically determined average dynamical-mass-to-stellar-mass ratio for *z* *=* 2.0–2.6 galaxies from ref. ^30^, $\log ({M}_{{\rm{dyn}}}/{M}_{{\rm{stellar}}})=-{0.38}_{-0.11}^{+0.11}$. This results in a stellar mass of $\log ({M}_{\text{star}}/{M}_{\odot })={11.39}_{-0.48}^{+0.52}\,{M}_{\odot }$.

As a check on the stellar mass, we also convert the integrated CO (3-2) flux into a CO line luminosity, *L*′_CO_, using the standard formula from ref. ^68^: ${L}_{\text{CO}}^{{\prime} }=3.25\times {10}^{7}\frac{{S}_{\text{CO}}{R}_{13}{D}_{{\rm{L}}}^{2}}{(1+z){\nu }_{\text{rest}}^{2}}\,{\rm{K}}\,{\rm{k}}{\rm{m}}\,{{\rm{s}}}^{-1}\,{{\rm{p}}{\rm{c}}}^{2}$, in which *S*_CO_ is the CO line flux in Jy km s^−1^, *D*_L_ is the luminosity distance in Mpc, *z* is the redshift and *ν*_rest_ is the rest frequency of the line in GHz. *R*_13_ is the CO (1-0)/CO (3-2) brightness temperature ratio such that *L*_CO_ is referred to the CO (1-0) line. We adopt *R*_13_ = 0.97, a typical value for quasars^69^, with which we find *L*′_CO_ = 6.91 × 10^10^ K km s^−1^ pc^2^. We then convert this to a molecular gas mass using the CO–H_2_ conversion factor, *α*_CO_, which we take as 4.36 *M*_⊙_ (K km s^−1^ pc^2^)^−1^ (refs. ^70–72^) with a 30% uncertainty. This results in a total molecular gas mass of log(*M*_H2_/*M*_⊙_) = 11.48 ± 0.13.

Combining the molecular gas mass and stellar mass leads to a molecular gas fraction of 0.55, consistent with gas fractions of massive star-forming galaxies at *z* ≈ 2 (ref. ^71^). The baryonic fraction, (*M*_stellar_ + *M*_H2_)/*M*_dyn_, is then 0.93, indicating little dark matter within the effective radius of the host galaxy, also consistent with deep, spatially resolved observations of *z* = 2 star-forming galaxies^73–75^. Therefore, if we would have made the assumption that the dynamical mass is entirely composed of the stellar and molecular gas mass, we would have arrived at log(*M*_stellar_) = 11.45, which is completely consistent with the stellar mass derived from the dynamical-to-stellar-mass ratio.

## Online content

Any methods, additional references, Nature Portfolio reporting summaries, source data, extended data, supplementary information, acknowledgements, peer review information; details of author contributions and competing interests; and statements of data and code availability are available at 10.1038/s41586-024-07053-4.

## Source data

Source Data Fig. 1

Source Data Fig. 2

Source Data Fig. 3

## Figures and Tables

**Extended Data Table 1:**
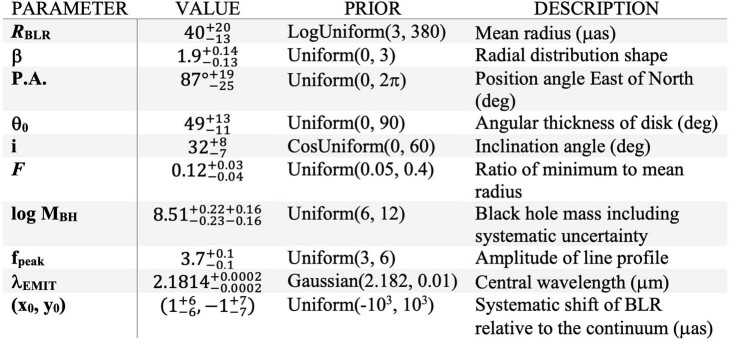
BLR model parameters and fit values

**Extended Data Table 2:**
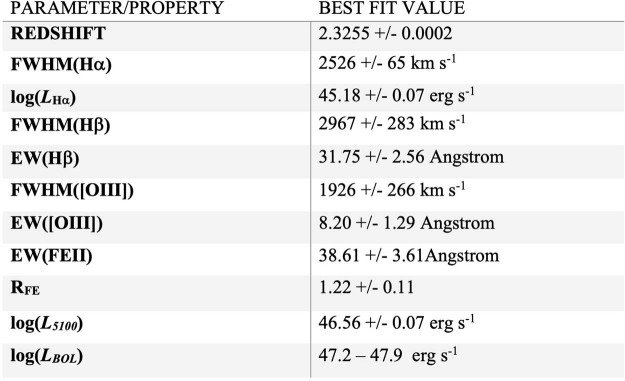
TripleSpec spectral decomposition

## Data Availability

The GRAVITY+ data used in this study are publicly available on the ESO archive (https://archive.eso.org/eso/eso_archive_main.html) under programme ID 110.2427. The NOEMA and APO/TripleSpec data are available from the corresponding author on request. Source data are provided with this paper.

## Code availability

The GRAVITY data-reduction pipeline is publicly available on the ESO webpage (https://www.eso.org/sci/software/pipelines/). GILDAS is publicly available on the IRAM webpage (https://www.iram.fr/IRAMFR/GILDAS/). astropy, matplotlib, emcee, dynesty, numpy and scipy are all available through the Python Package Index (https://pypi.org). The custom photocentre fitting and BLR modelling packages are available on request from the corresponding author.
